# Reduction in Interferon-Stimulated Genes Contributes to High-Yield Production of Influenza Virus in Suspension MDCK Cells

**DOI:** 10.3390/vaccines12030287

**Published:** 2024-03-09

**Authors:** Qi Wang, Jian Luo, Beibei Li, Qian Ye, Wenting Xu, Feixia Gao, Linting Zhou, Wenyue Lu, Wen-Song Tan, Xiuling Li

**Affiliations:** 1 Department of Virus and Vaccine, Shanghai Institute of Biological Products, Shanghai 200052, China; 2 State Key Laboratory of Bioreactor Engineering, East China University of Science and Technology, Shanghai 200237, China

**Keywords:** influenza virus, suspension MDCK cells, vaccines, interferon-stimulated genes, high-yield production

## Abstract

Compared with the traditional vaccine produced in embryonated chicken eggs, cell-based manufacturing represented by the Madin–Darby canine kidney (MDCK) cell line has a larger production scale and reduces the risk of egg shortage in a pandemic. Establishing a culture system that enables high production of the influenza virus is a key issue in influenza vaccine production. Here, a serum-free suspension culture of MDCK (sMDCK) cells was obtained from adherent MDCK (aMDCK) cells by direct adaptation. Viral infection experiments showed that viral yields of influenza A/B virus in sMDCK cells were higher than in aMDCK cells. Transcriptome analysis revealed that numerous interferon-stimulated genes (ISGs) exhibited reduced expression in sMDCK cells. To further clarify the mechanism of high viral production in sMDCK cells, we demonstrated the antiviral role of RIG-I and IFIT3 in MDCK cells by knockdown and overexpression experiments. Furthermore, suppression of the JAK/STAT pathway enhances the viral accumulation in aMDCK cells instead of sMDCK cells, suggesting the reduction in the JAK/STAT pathway and ISGs promotes viral replication in sMDCK cells. Taken together, we elucidate the relationship between the host innate immune response and the high viral productive property of sMDCK cells, which helps optimize cell production processes and supports the production of cell-based influenza vaccines.

## 1. Introduction

Seasonal influenza viruses are important respiratory pathogens that have a significant impact on human health and economic development. To date, the most effective means of preventing influenza is vaccination. Although the traditional influenza vaccines in chicken embryos are still predominantly used worldwide, the supply of embryonated eggs and egg-adaptive mutations in vaccine virus strains affects vaccine production and efficacy [1,2,3]. In contrast, cell-based vaccines have several advantages over egg-based technologies. Firstly, cell expansion was performed in automated bioreactors. Thus, this technology greatly shortens the production cycle of vaccines and reduces the risk of microbial contamination [4]. Secondly, vaccine strains produce fewer HA mutations in mammalian cells than in eggs, which increases the antigenic match of vaccines and circulating virus strains [5]. Moreover, cell-based vaccines can reduce the risk of egg allergies. In recent years, seasonal influenza vaccines and pandemic influenza vaccines based on different cell substrates have been approved for marketing in different countries and regions, providing important support for the prevention and control of influenza.

Cell lines including Madin–Darby canine kidney (MDCK), PER.C6, and Vero are used in vaccine research and development [4,6,7,8]. MDCK cells belong to epithelioid cells and contain both α-2,3- and α-2,6-linked sialic acid (SA) receptors, which are sensitive to various influenza viruses. As MDCK cells normally exhibit adhesive growth and need serum in vitro culture, large-scale production is limited [9,10,11]. There are several research opportunities for improving virus yields in MDCK culture processes, one of which is suspension culture. MDCK suspension cells obtained by serum-free adaption culture have a faster growth rate, allowing for mass production, which has been reported to produce influenza vaccines [12,13,14,15,16,17]. However, most research focuses on cell growth and metabolic regulation, and limited studies have examined the mechanism of cell immune response during virus production [16,18].

Innate immune response in mammalian cells restricts viral propagation. For the influenza virus, Retinoic acid-inducible gene I (RIG-I, encoded by the *DDX58* gene) is the major pathogen recognition receptor (PRR) of the cytoplasm to induce the production of type I interferon (IFN-α/β) and pro-inflammatory cytokines [19]. It has been reported that canine RIG-I suppresses the replication of the canine influenza virus (CIV) in adherent MDCK (aMDCK) cells [20]. The secreted IFN-α/β activates the JAK/STAT signaling pathway, leading to the induction of hundreds of IFN-stimulated genes (ISGs) including ISG15, MX1, PKR, OAS1, IFI44, and IFITM3 [21,22,23,24,25]. The ATCC-modified STAT1 knock-out aMDCK cells enhanced the viral titer of IAV by thirty times. The antiviral role of IRF7 has been validated by constructing an IRF7 knock-out MDCK cell [26]. RSAD2 has also been confirmed to inhibit IAV production in aMDCK cells [27]. However, only a few ISGs have been well studied in MDCK cells during influenza infection. Therefore, the systematic comparison of the host innate immune responses of different MDCK cells to the influenza virus still needs to be further investigated.

In this study, we established a serum-free MDCK suspension cell line, named sMDCK cells, which had higher viral yields than aMDCK cells. Transcriptome analysis revealed that compared with aMDCK cells, ISGs in sMDCK cells were significantly downregulated after viral infection. Furthermore, canine RIG-I and IFN-induced protein with tetratricopeptide repeats 3 (IFIT3) were found to be responsible for the reduced antiviral response in sMDCK cells. Our work explains the mechanism of high-yield production of the influenza virus in suspension MDCK cells from a new perspective, which will help us better understand the host immune regulation of MDCK cells against viral infection and provide support for the production of cell-based influenza vaccines.

## 2. Materials and Methods

### 2.1. Cells and Virus

The adherent MDCK (aMDCK) cells (CCL-34, ATCC) were cultured in Dulbecco’s Modified Eagle Medium (DMEM) (Gibco, New York, NY, USA) with 10% fetal bovine serum (FBS) (Gibco, New York, NY, USA) at 37 °C, 5% CO_2_. The suspension MDCK (sMDCK) cells were adapted from aMDCK cells and cultured in MDCK serum-free medium (Bio-engine, Shanghai, China) at 37 °C, 5% CO_2_, under 120 rpm. The cell morphology was observed under microscopy (OLYMPUS, Tokyo, Japan). Influenza virus A/Puerto Rico/8/34 (H1N1), designated PR8, and influenza virus B/Austria/1359417/2021(BVR-26), designated BVR-26, were stored at −80 °C in our laboratory.

### 2.2. Sialic Acids (SAs) Detection Assay

The α-2,6-SA and α-2,3-SA were detected using the DIG glycan differentiation kit (Roche, Mannheim, Germany). Briefly, aMDCK and sMDCK cells were stained with DIG-labeled antibodies for 1 h after washing and centrifugation. The cells were washed with PBS containing 5% FBS and incubated with anti-DIG FITC for 1 h at 37 °C [28]. The cell surface SA was analyzed using flow cytometry.

### 2.3. Plasmids and Molecular Cloning

The canine RIG-I and IFIT3 genes were amplified from aMDCK cell cDNA and then cloned into pCMV vectors with an N-terminal 3xFLAG epitope (pCMV-3Flag) to generate pCMV-3Flag-RIG-I and pCMV-3Flag-IFIT3. The EGFP gene was amplified from pCDNA-EGFP and cloned into pCMV-3Flag to generate pCMV-3Flag-EGFP. Primers for amplification are shown in Table 1.

### 2.4. Cell Culture Infection and Transfection

aMDCK cells were seeded in T75 flasks (Corning, New York, NY, USA) and sMDCK cells were seeded in 125 mL shake flasks (Corning, New York, NY, USA) before infection. For aMDCK cells, 90–100% confluent cells were washed twice with phosphate-buffered saline (PBS) (Gibco, New York, NY, USA). For sMDCK cells, cells were harvested by centrifugation at 1000 rpm for 5 min, washed with PBS, and resuspended in serum-free medium (at a density of 2 × 10^6^ cells/mL). Then aMDCK and sMDCK cells were infected with the PR8 virus at a multiplicity of infection (MOI) of 0.001 in the presence of 2 μg/mL N-tosyl-L-phenylalanine chloromethyl ketone (TPCK)-trypsin (Sigma-Aldrich, Saint Louis, MO, USA) to promote viral amplification. Cell supernatants were collected for HA assay and Western blot, and the precipitated cells were collected for Western blot and RT-qPCR.

For overexpression experiments, plasmids expressing Flag-RIG-I, Flag-IFIT3, or Flag-EGFP were transfected into sMDCK cells using Lipofectamine™ 3000 (Invitrogen, Carlsbad, CA, USA) for 24 h and then inoculated with the PR8 virus at an MOI of 0.001 in the presence of 2 μg/mL TPCK-trypsin. After 24, 48, and 72 h of infection, cell supernatants were collected for TCID_50_ assay.

### 2.5. Determination of Virus Titer

Total virus titer was measured by HA assay [29]. Briefly, the virus was serially diluted 2-fold with PBS and mixed with 50 μL 1% chicken red blood cells in U-bottom 96-well microplates. The mixture was incubated for 30 min at room temperature. HA titers were determined as Log_10_HA units per test volume (Log_10_HAU/50μL).

The cell-specific virus yield was calculated as described previously [30]. Specifically, chicken red blood cells were set at 2 × 10^7^ cells/mL before HA assay. The specific virus yield was defined as the product of the highest virus titer and chicken red blood cell density divided by the maximum live cell density.

For viral TCID_50_ assay, aMDCK cells were seeded in 96-well plates (2 × 10^4^ cells/well) and inoculated with 10-fold dilutions of samples for 1 h at 37 °C. Then cells were washed and added to DMEM containing 2 μg/mL TPCK-trypsin for 72 h at 35 °C. Culture supernatants were aspirated for HA assay. TCID_50_ was calculated using the Reed–Muench method [31].

### 2.6. Western Blotting Assay

aMDCK cells were collected after 0.25% trypsin enzyme (Gibco, USA) treatment and counted. aMDCK and sMDCK cells were collected by centrifugation at 1000 rpm for 5 min, and washed with PBS. After centrifugation, 2 × 10^6^ aMDCK or sMDCK cells were added to 200 μL cell lysis buffer (Beyotime, Shanghai, China) for 30 min on ice. The supernatants were collected after centrifuging at 12,000 rpm for 5 min, and then SDS PAGE Loading Buffer (Takara, Tokyo, Japan) was added. Samples were boiled for 10 minutes and separated by SDS gel electrophoresis. After transfer to PVDF (Millipore, Bedford, MA, USA), the membrane was blocked for 1 h at room temperature and then incubated overnight at 4 °C with primary antibodies. The following primary antibodies were used for detection: GAPDH (Transgene, Beijing, China), NS1 (Santa Cruz, CA, USA), RIG-I (Santa Cruz, CA, USA), IFIT3 (ABclonal, Wuhan, China), MAVS (Santa Cruz, CA, USA), and Flag (GenScript, Nanjing, China). After incubating with secondary antibody for 1 h, signal detection was performed using Azure Imaging Systems (Azure Biosystem, Dublin, CA, USA).

### 2.7. RT-qPCR

Total RNAs were extracted using TRIzol (Invitrogen, Carlsbad, CA, USA). Then, 1 μg of RNA was reverse transcribed to cDNA using cDNA Synthesis kit (Vazyme, Nanjing, China). qPCR was performed using SYBR qPCR Master Mix (Vazyme, Nanjing, China). All samples were performed in triplicate. The results were normalized to canine GAPDH mRNA. The relative expressions of genes were determined using the ΔΔCt method. The primer sequences used in RT-qPCR are listed in Table 2.

### 2.8. RNA Interference Assay

Small interfering RNAs (siRNAs) targeting canine RIG-I (siRIG-I) and IFIT3 (siIFIT3) were synthesized by Sangon (Shanghai, China). The sequences of siRIG-I were 5′-GGUACAAAGUUGCAGGUAUTT-3′(sense), and 5′-AUACCUGCAACUUUGUACCTT-3′(antisense). The sequences of siIFIT3 were 5′-GCAUCGGAAUCCCUUCCAATT-3′(sense), and 5′-UUGGAAGGGAUUCCGAUGCTT-3′(antisense). aMDCK cells were seeded in a 24-well plate (2 × 10^5^ cells/well) and transfected with 100 nM siRNA or non-targeting control siRNA (siNC) using Lipofectamine™ RNAiMAX (Invitrogen, Carlsbad, CA, USA). The aMDCK cells were then infected with the PR8 virus (MOI = 0.001) after 36 h post-transfection. Supernatants were collected at 24, 48, and 72 h to determine viral titers.

### 2.9. JAK Inhibition

MDCK cells were treated with 5μM Pyridone 6 [2-(1,1-Dimethylethyl)-9-fluoro-3,6-dihydro-7H-benz[h]-imidaz[4,5-f] isoquinolin-7-one] (Beyotime, Shanghai, China) [32] or DMSO as a control for 24 h and assayed for ISG expression by RT-qPCR. P6-treated or DMSO-treated MDCK cells were infected with the PR8 virus at an MOI of 5 for 8 h, and then virus accumulation was detected by RT-qPCR.

### 2.10. RNA-seq and Data Analysis

Total RNA samples were isolated using TRIzol reagent. Briefly, aMDCK cells were washed twice with PBS and added to 1 mL TRIzol reagent per 5 × 10^6^ cells. sMDCK cells were counted and harvested by centrifugation at 1000 rpm for 5 min and then washed with PBS. After centrifuging, 1 mL TRIzol reagent was used to lysis 5 × 10^6^ sMDCK cells. The processed cell samples were transferred to 2 mL threaded cryopreservation tubes and snap-frozen in liquid nitrogen for 30 min. The labeled samples were then submitted to Shanghai Zhongke New Life Biotechnology Co., Ltd. (Shanghai, China) for RNA sequencing.

After removing adapter sequences and low-quality reads from raw data, clean reads were aligned to the reference genome of Canis lupus familiaris (HISAT2 index: Ensembl CanFam3.1 genome_tran) using HISAT2 software (version 2.2.0) [33]. The screening of Differential expression genes (DEGs) used the following criteria: |log2fold change| > 1 and *p*-value < 0.05. Hierarchical clustering, volcano plot, Gene Ontology (GO) function enrichment, and KEGG pathway enrichment analysis of DEGs were performed by R package software package (version 4.1.0) [34,35] to compare the differences between aMDCK and sMDCK cells.

### 2.11. Statistical Analysis

Statistical analyses were performed using GraphPad Prism (version 8, La Jolla, CA, USA). All experiments contained three biological replicates and *p* value < 0.05 was considered significant.

## 3. Results

### 3.1. Biological Characteristics Analysis of sMDCK Cells

**Figure 1:**
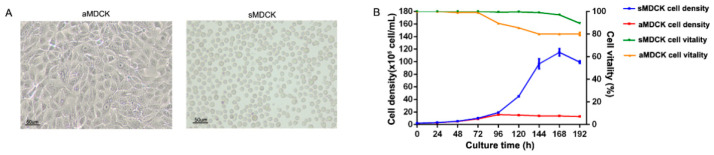
Biological characteristics of sMDCK cells. (**A**) Cellular morphology of aMDCK and sMDCK cells. (**B**) Cell density and cell vitality curves of aMDCK and sMDCK cells. aMDCK cells and sMDCK cells were seeded at equal densities (2 × 10^5^ cells/mL) in T75 flasks or 125 mL shake flasks, respectively. Cell density and cell viability were monitored every 24 h.

To obtain suspension MDCK cells, we used the direct adaptation method by culturing aMDCK cells in a serum-free medium. Following 15 generations of serum-free suspension culture, sMDCK cells were uniform in morphology, presenting single-cell suspension growth (Figure 1A). The results of cell growth curves showed that after inoculating different MDCK cells with the same cell density, sMDCK cells reached the maximum specific growth rate at 120 h, and the cell density reached the maximum value of 11.5 × 10^6^ cells/mL at 168 h, with cell viability consistently exceeding 90% during the process of cultivation (Figure 1B). aMDCK cells reached the maximum specific growth rate at 72 h of cultivation. After 96 h of cultivation, the cell density reached the maximum value of 1.58 × 10^6^ cells/mL, and the cell viability was 90%. After 192 h of cultivation, the cell survival rate was approximately 80%. Thus, the highest cell density, maximum specific growth rate, and cell viability of sMDCK cells cultured in serum-free suspension culture were significantly higher than those of aMDCK cells cultured in serum-containing adherent culture, which was conducive to achieving high-density cultivation of MDCK cells.

The infection of the influenza virus may be closely related to the abundance of SA receptor expression on the surface of host cells [36,37]. SA receptors were detected at high levels in both cells using flow cytometry (Figure S1). The expression rates (positive cells/total cells) of α-2,6 and α-2,3-SA receptors on the surface of aMDCK cells were 99.74% and 98.21%, respectively, and the expression rates of two receptors on the surface of sMDCK cells were 99.03% and 99.00%, respectively. There was no significant difference in the expression levels of these two receptors on the surface of two cell lines, suggesting that the serum-free suspension domestication process did not affect the expression of SA receptors on the surface of MDCK cells.

### 3.2. Transcriptomic Analysis of sMDCK vs. aMDCK Cells

**Figure 2:**
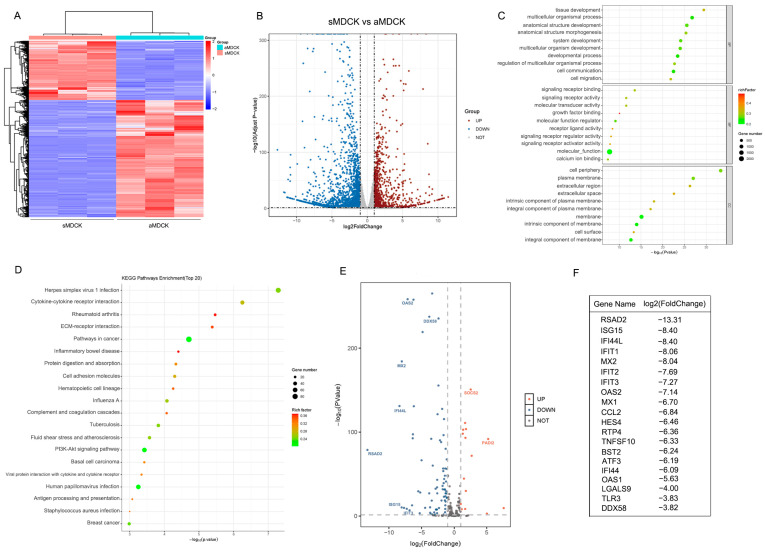
Transcriptome analysis of sMDCK vs. aMDCK cells. (**A**) Heatmap of differentially expressed genes (DEGs) between aMDCK and sMDCK cells. (**B**) Volcano plot shows DEGs between aMDCK and sMDCK cells. (**C**) Gene ontology (GO) analysis of DEGs between aMDCK and sMDCK cells. These genes were classified into different categories according to biological processes, cellular components, and molecular function. (**D**) Top 20 Kyoto Encyclopedia of Genes and Genomes (KEGG) pathway analyses of downregulated genes. (**E**) Volcano plot shows differentially expressed interferon-stimulated genes (ISGs) between aMDCK and sMDCK cells. (**F**) Top 20 significantly downregulated ISGs.

To compare the differential gene expression between aMDCK and sMDCK cells, high-throughput RNA sequencing was performed. A DESeq2 analysis using padj < 0.05 and |log2(foldchange)| > 1 as the screening criteria identified a total of 3097 differentially expressed genes (DEGs). The hierarchical clustering of DEGs revealed the samples clustered into two groups (Figure 2A). The volcano plot showed that 2057 DEGs were downregulated and 1040 DEGs were upregulated in the sMDCK relative to aMDCK (Figure 2B). GO analysis including three parts—biological processes, cellular components, and molecular function—was carried out to determine the main biological function of these DEGs. The top 30 regulated GO terms are shown in the GO bubble chart (Figure 2C). Within the biological processes, DEGs were mainly engaged in the development process including tissue development (GO: 0009888) and the multicellular organismal process (GO: 0007275). In the cellular components group, the cell periphery (GO: 0071944) and plasma membrane (GO: 0005886) were enriched, which suggested the morphology and adhesion of aMDCK were altered during the suspension culture of sMDCK cells. The top 20 downregulated KEGG pathways are shown in Figure 2D. In addition to the pathway of cell adhesion molecules, it was shown that downregulated genes involved in the process of viral infection, especially the pathway of influenza A virus infection, indicated that the antiviral state may have changed between aMDCK and sMDCK cells.

To further analyze gene expression levels associated with antiviral IFN response, ISGs were screened and shown with a volcano plot (Figure 2E). Among the top 20 downregulated ISGs, some genes including RSAD2, ISG15, and Mx1 have been reported to exert anti-influenza virus effects [27,38,39] (Figure 2F). In addition, there were several upregulated ISGs in sMDCK cells. For example, suppressor of cytokine signaling 2 (SOCS2) has been reported to play a negative role in activating the JAK/STAT signaling pathway [40,41]. In conclusion, the antiviral innate immune pathway was significantly reduced in sMDCK cells.

### 3.3. Validation of Gene Expression by RT-qPCR

**Figure 3:**
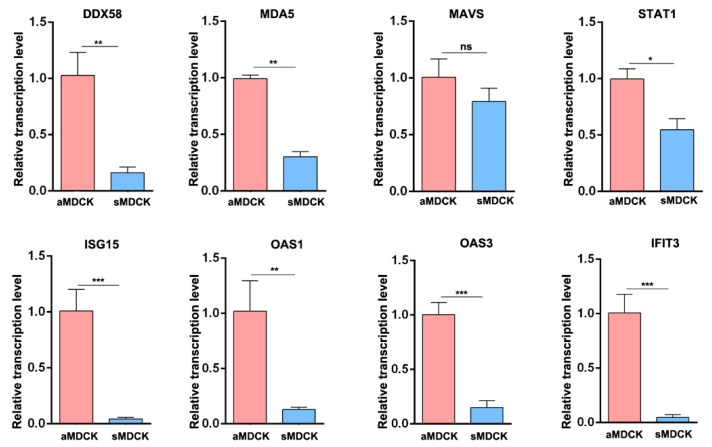
Transcription level verified by RT-qPCR. Total RNAs from aMDCK and sMDCK cells were extracted and RT-qPCR was carried out to examine the expression of genes including DDX58 (RIG-I), MDA5, MAVS, STAT1, ISG15, OAS1, OAS3, and IFIT3. The experiments were repeated three times independently. The mRNA level of aMDCK cells was set as 1. Canine GAPDH was used as the reference gene. * *p* < 0.05, ** *p* < 0.01, *** *p* < 0.001 (Student’s *t*-test). ns indicates no significance.

Subsequently, we used RT-qPCR to confirm the transcriptomic data. It showed that the mRNA levels of ISGs including DDX58 (RIG-I), MDA5, STAT1, ISG15, OAS1, OAS3, and IFIT3 were significantly downregulated in sMDCK cells (Figure 3). There was no significant difference in the expression of the adaptor protein MAVS, which was consistent with the analysis of RNA sequencing.

### 3.4. Viral Yields of Influenza A/B Virus Were Higher in sMDCK Cells Than in aMDCK Cells

**Figure 4:**
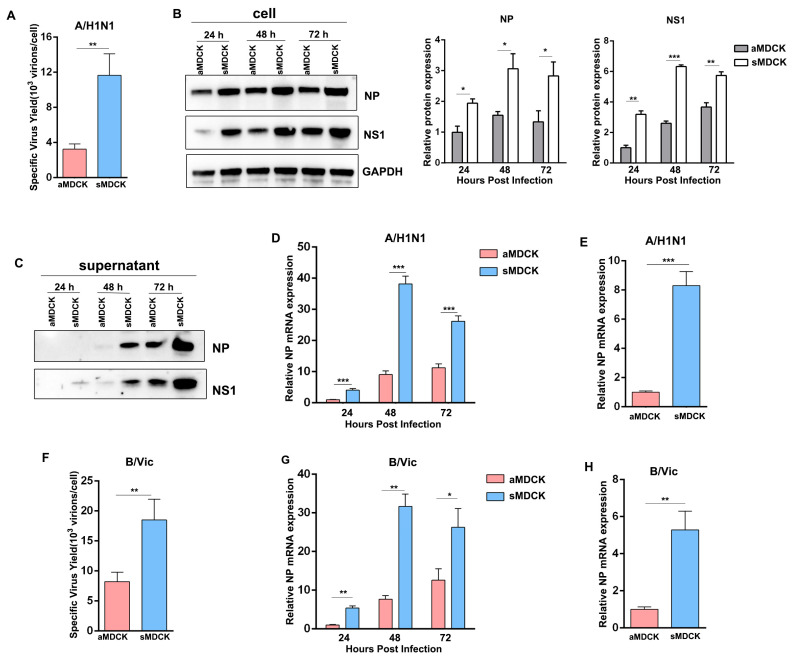
Viral yields of influenza A/B virus were higher in sMDCK cells than in aMDCK cells. (**A**) Specific virus yield of aMDCK and sMDCK cells after PR8 virus infection. NP and NS1 expression in cell lysate (**B**) and supernatants (**C**) were detected in aMDCK and sMDCK cells infected with PR8 virus (MOI = 0.001) at 24, 48, and 72 h post-infection (hpi) by Western blot. (**D**) Relative NP accumulations of PR8 virus in the cell lysate at 24, 48, and 72 hpi were quantified by RT-qPCR. (**E**) Relative NP accumulations in cell lysate were detected in aMDCK and sMDCK cells infected with PR8 virus (MOI = 5) at 8 hpi by RT-qPCR. (**F**) Specific virus yield of aMDCK and sMDCK cells after BVR-26 infection. (**G**) Relative NP accumulations of BVR-26 in the cell lysate at 24, 48, and 72 hpi were quantified by RT-qPCR. (**H**) Relative NP accumulations in cell lysates were detected in aMDCK and sMDCK cells infected with BVR-26 (MOI = 5) at 8 hpi by RT-qPCR. Canine GAPDH was used as the reference gene. * *p* < 0.05, ** *p* < 0.01, *** *p* < 0.001 (Student’s *t*-test). ns indicates no significance.

We hypothesized that the attenuated antiviral response enhanced viral production in sMDCK cells. Thus, we conducted viral infection experiments to verify whether viral titer was elevated in sMDCK cells. Results showed that the specific virus yield of the PR8 virus was significantly higher in sMDCK cells than in aMDCK cells (Figure 4A). To compare the viral production in different MDCK cells at the same time point, the PR8 virus was infected in aMDCK and sMDCK cells. Western blot results demonstrated that viral protein NP and NS1 in sMDCK cells and culture supernatant both increased at 24 h, 48 h, and 72 h post-infection (Figure 4B,C). The relative accumulation of viruses in sMDCK cells also increased compared with aMDCK cells (Figure 4D). In addition, considering the different growth rates of aMDCK and sMDCK cells, we compared viral production at 8 h post-infection. Results showed that after single-cycle infection, virus accumulation in sMDCK cells was still higher than the yield from aMDCK cells (Figure 4E).

To further confirm the above results, we selected the B/Vic vaccine strain (BVR-26), which is also an important component of seasonal influenza vaccines, for virus infection experiments. We infected the two MDCK cell lines with BVR-26 and found the specific virus yield and virus accumulation were significantly higher in sMDCK cells (Figure 4F–H). These results indicated that sMDCK cells had higher efficiencies in producing influenza vaccines than aMDCK cells.

### 3.5. Transcriptomic Analysis of sMDCK vs. aMDCK after Viral Infection

**Figure 5:**
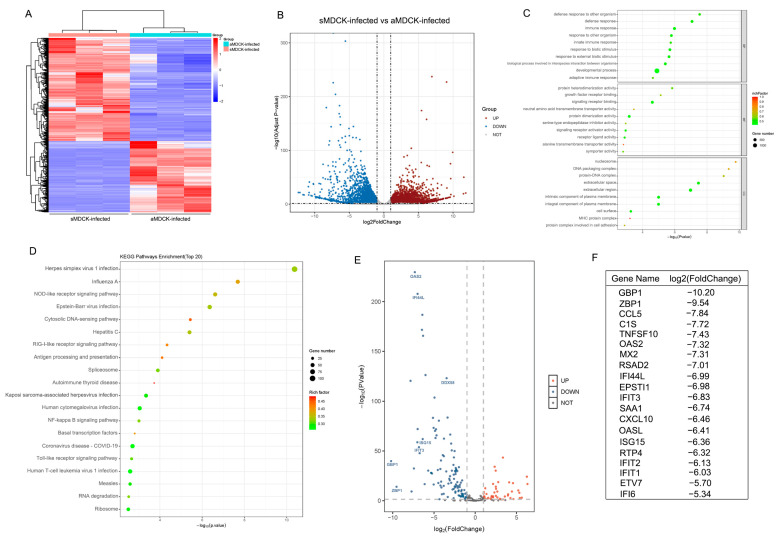
Transcriptomic analysis of sMDCK vs. aMDCK cells after viral infection. Heatmap (**A**) and volcano plot (**B**) of DEGs from sMDCK vs. aMDCK cells after viral infection. (**C)** GO analysis of DEGs from sMDCK vs. aMDCK cells after viral infection. (**D**) Top 20 KEGG pathway analyses of downregulated genes. (**E**) Volcano plot shows differentially expressed ISGs between aMDCK and sMDCK cells. (**F**) Top 20 significantly downregulated ISGs.

To analyze the mechanisms of high virus production in sMDCK cells, transcriptome analyses were performed to compare sMDCK and aMDCK cells after PR8 virus infection. From the Hierarchical clustering, these samples were divided into two groups (Figure 5A). In total, we identified 7835 DEGs, including 4634 upregulated and 3201 downregulated (Figure 5B). The top 30 regulated GO terms are shown in the GO bubble chart (Figure 5C). In biological processes, DEGs were mainly engaged in immune responses including the innate immune system (GO:0045087) and defense response (GO:0006952), suggesting that the replication differences of the virus in two cell lines are related to the innate immune pathway. The top 20 downregulated KEGG pathways are shown in Figure 5D. Pathway enrichment analysis showed that DEGs participate in influenza A and RIG-I-like receptor signaling pathways, which provides further evidence for the regulatory role of the interferon response in viral replication between aMDCK and sMDCK cells. The volcano plot of differentially expressed ISGs is shown in Figure 5E. A large number of ISGs were significantly downregulated in sMDCK cells after viral infection (Figure 5F).

### 3.6. The Induced ISG in sMDCK Cells Significantly Decreases Compared with aMDCK Cells

**Figure 6:**
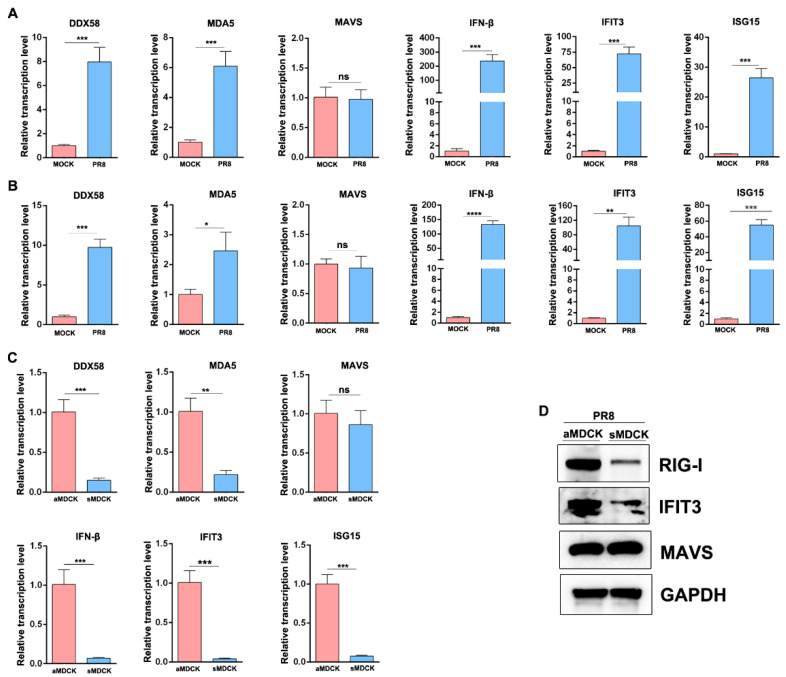
ISGs were decreased in sMDCK cells after viral infection. aMDCK and sMDCK cells were infected with the PR8 virus at an MOI of 0.01 for 48 h. Transcription levels of DDX58 (RIG-I), MDA5, MAVS, IFN-β, IFIT3, and ISG15 in aMDCK cells (**A**) and sMDCK cells (**B**) were detected by RT-qPCR. (**C**) Relative transcription levels of DDX58, MDA5, MAVS, IFN-β, IFIT3, and ISG15 in aMDCK cells and sMDCK cells. Canine GAPDH was used as the reference gene. * *p* < 0.05, ** *p* < 0.01, *** *p* < 0.001, **** *p* < 0.0001 (Student’s *t*-test). ns indicates no significance. (**D**) Western blot was performed to detect RIG-I, IFIT3, MAVS, and GAPDH expressions in aMDCK and sMDCK cells after viral infection.

To confirm the differential expression of ISG in aMDCK and sMDCK following virus infection, mRNA levels of IFN-related genes were analyzed by RT-qPCR. Results showed that the virus induces IFN-β and ISGs (DDX58, MDA5, IFIT3, ISG15) expression in both aMDCK and sMDCK cells (Figure 6A,B). However, when comparing ISG induction between aMDCK and sMDCK cells after viral infection, we found that virus-induced ISG expressions were significantly decreased in sMDCK cells (Figure 6C), consistent with our transcriptomic analysis.

In addition to the mRNA level, we also tested protein expressions by Western blot (Figure 6D). RIG-I and IFIT3 expression were significantly reduced in sMDCK cells. Furthermore, we found that the expression of MAVS was not changed, indicating that the IFN signaling pathway in sMDCK cells was weakened but not imbalanced.

### 3.7. Reduction in the Induced RIG-I and IFIT3 in sMDCK Cells Promotes Viral Production

**Figure 7:**
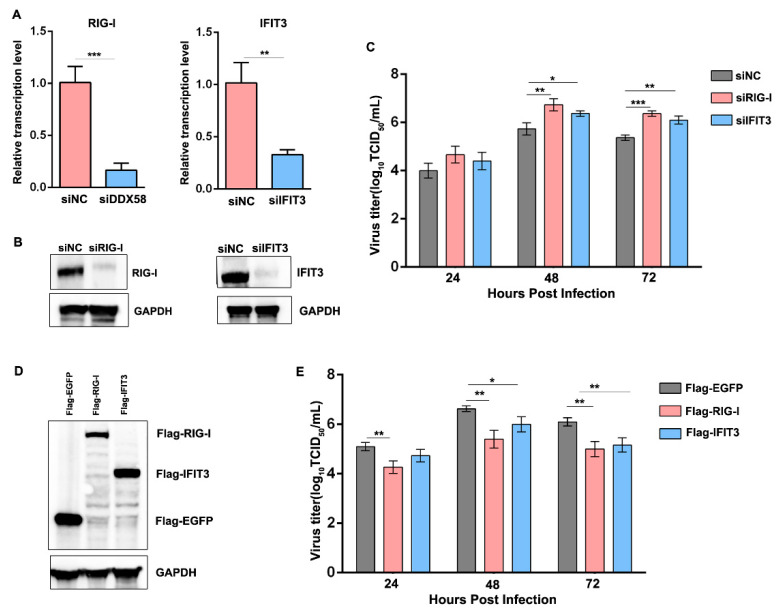
RIG-I and IFIT3 inhibit viral infection in MDCK cells. (**A**) aMDCK cells were transfected with siRIG-I, siIFIT3, or siNC for 36 h. Cells were harvested and transcription levels of RIG-I and IFIT3 were detected by RT-qPCR. Canine GAPDH was used as the reference gene. ** *p* < 0.01, *** *p* < 0.001 (Student’s *t*-test). ns indicates no significance. (**B**) Western blot detection of RIG-I and IFIT3. (**C**) aMDCK cells were transfected with siRIG-I, siIFIT3, or siNC for 36 h and then infected with PR8 virus at an MOI of 0.001. Viral titers were determined by a TCID_50_ assay at 24, 48, and 72 hpi. * *p* < 0.05, ** *p* < 0.01, *** *p* < 0.001 (Student’s *t*-test). (**D**) sMDCK cells were transfected with pCMV-3Flag-RIG-I, pCMV-3Flag-IFIT3, and pCMV-3Flag-EGFP for 36 h. Western blot was performed to detect RIG-I, IFIT3, and EGFP expressions. (**E**) sMDCK cells were transfected with pCMV-3Flag-RIG-I, pCMV-3Flag-IFIT3, and pCMV-3Flag-EGFP for 36 h and then infected with the PR8 virus at an MOI of 0.001. Viral titers were determined by a TCID_50_ assay at 24, 48, and 72 hpi. * *p* < 0.05, ** *p* < 0.01, *** *p* < 0.001 (Student’s *t*-test).

Collectively, our data indicated that virus-infected sMDCK cells induced low levels of ISG protein expression, such as RIG-I and IFIT3. We then explored whether the reduced canine RIG-I and IFIT3 enhanced viral replication in sMDCK cells. aMDCK cells were transfected with siNC, siRIG-I, or siIFIT3 and knockdown was confirmed by RT-qPCR and Western blot (Figure 7A,B). We also confirmed the effects of RIG-I and IFIT3 by infecting knockdown MDCK cells. The viral titers significantly increased in the supernatant of RIG-I and IFIT3 knockdown cells at 48 and 72 hpi compared with siNC control aMDCK cells (Figure 7C).

On the other hand, Flag-EGFP, Flag-RIG-I, and Flag-IFIT3 were, respectively, overexpressed in sMDCK cells and then infected with the PR8 virus (Figure 7D). The viral titers significantly decreased in the supernatant of RIG-I and IFIT3 overexpression sMDCK cells at different time points after viral infection (Figure 7E). Taken together, we concluded that the reduction in the induced canine RIG-I and IFIT3 in sMDCK cells inhibits antiviral immune response, leading to promoted viral production.

### 3.8. Inhibition of JAK/STAT Pathway Increased the Production of IAV in aMDCK Cells Instead of sMDCK Cells

**Figure 8:**
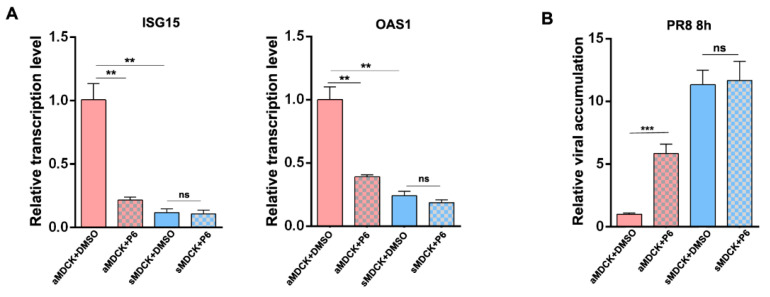
Reduced ISG promotes viral replication in sMDCK cells. (**A**) P6-treated aMDCK and sMDCK cells were harvested and transcription levels of ISG15 and OAS1 were detected by RT-qPCR. (**B**) Effect of P6 addition in aMDCK and sMDCK cells on viral accumulation. Canine GAPDH was used as the reference gene. ** *p* < 0.01, *** *p* < 0.001 (Student’s *t*-test). ns indicates no significance.

To assess the role of more downregulated ISGs in sMDCK cells, we tested the effect of the suppression of JAK/STAT signaling by adding pyridone 6 (P6) during infection. P6 is a pan-JAK inhibitor and was verified to inhibit ISG expression through competitively binding to JAK1, JAK2, JAK3, and Tyk2 [32,42,43,44]. ISG expression levels were examined in P6-treated aMDCK and sMDCK cells. The results showed that the expression levels of ISG15 and OAS1 were significantly reduced after P6 treatment of aMDCK cells (Figure 8A,B). P6-treated aMDCK cells showed increased viral accumulation compared with the DMSO control group, while the viral replication in sMDCK cells was not affected by P6 (Figure 8B). Thus, we speculated that the difference in susceptibility of MDCK cells to influenza virus infection is due to different levels of ISG expression, with sMDCK cells having a lower level of ISG expression.

## 4. Discussion

With the increasing demand for global influenza prevention and control requirements and influenza vaccine products, the efficient, stable, and controllable cell-based production platform has become an important direction for influenza vaccine production. The WHO recommends using cells as the substrate for influenza vaccine production and MDCK cells are the most commonly used cells. Compared to the traditional serum-containing adherent culture, serum-free suspension culture technology has the advantages of simple operation, easy scaling up, lower cost, and higher stability. Here, we successfully obtained sMDCK cells derived from aMDCK cells by serum-free suspension culture. We compared cell growth rates of the two cell lines and found the highest cell density and maximum specific growth rate of sMDCK cells were significantly higher than that of aMDCK cells, which was conducive to the realization of a high-density culture of sMDCK cells. In addition, the maximal viable cell density of sMDCK cells in our work was comparable to that of the suspension MDCK cells reported in the previous study [13]. SA receptors on the surface of MDCK cells were critical for influenza virus infection [37,45]. There was no significant difference in SA receptors of aMDCK and sMDCK cells. We performed transcriptome analysis by using high-throughput sequencing and found, except for genes encoding adhesion proteins, the expression of a large number of ISGs in sMDCK cells was significantly lower than that in aMDCK cells, indicating the potential advantages of sMDCK cells in virus amplification.

Improving viral yield has always been one of the most concerning issues in cell-based vaccine production. Our results demonstrated higher virus yields in sMDCK cells after IAV infection. Moreover, the influenza B virus vaccine strain BVR-26 was also confirmed to have increased the yield in sMDCK cells. To explain this observation, we conducted transcriptome analysis and found the innate immune system had been downregulated in sMDCK cells. We validated these reduced ISGs at the mRNA and protein levels. Interestingly, we also found that some ISGs were significantly upregulated, such as TRIM38. TRIM38 inhibits TLR3/4- and RIG-I–mediated IFN-β signaling in mouse macrophage cells and HEK293 cells [46]. TRIM38 also plays a negative role in inducing NF-κb in osteoclasts [47]. However, a recent study shows that TRIM38 positively regulates MDA5- and RIG-I-mediated signaling in HEK293 cells [48]. Therefore, the regulator role of TRIM38 and other ISGs needs to be studied in the future.

The mechanism underlying how the host’s innate response against IAV infection in MDCK cells was not fully understood. Zhou et al. analyzed mRNA expression profiles of the host genes in human epithelial cells infected with IAV and found antiviral factors participating in immune response, including OAS1, OAS2, IFI44, RIG-I, IFIT2, and IFIT3 [25]. IFIT3 is an antiviral factor for many viruses, including the adenovirus, dengue virus (DENV), Newcastle disease virus (NDV), and influenza virus [49,50,51,52,53]. The antiviral role of IFIT3 in IAV infection was validated in human lung epithelial (A549) cells [53]. Here, knockdown of IFIT3 enhances viral titer in aMDCK cells, while overexpression of IFIT3 inhibits viral replication in sMDCK cells, demonstrating the antiviral effect of IFIT3 in MDCK cells. In addition, RIG-I had also been confirmed to be involved in antiviral immune responses in MDCK cells, in line with the previously reported study [20]. The JAK/STAT pathway induced by IFN-I causes ISG production, which in turn inhibits viral infections [54]. The addition of the JAK inhibitor (P6) enhanced virus production in aMDCK cells but not sMDCK, suggesting reduced ISGs lead to high viral production in sMDCK cells. In addition, the JAK/STAT and NF-κB signaling pathways are also involved in the regulation of many inflammatory responses [55,56]. The subdued inflammatory response in sMDCK cells may be another reason for its high virus production [28].

## 5. Conclusions

**Figure 9:**
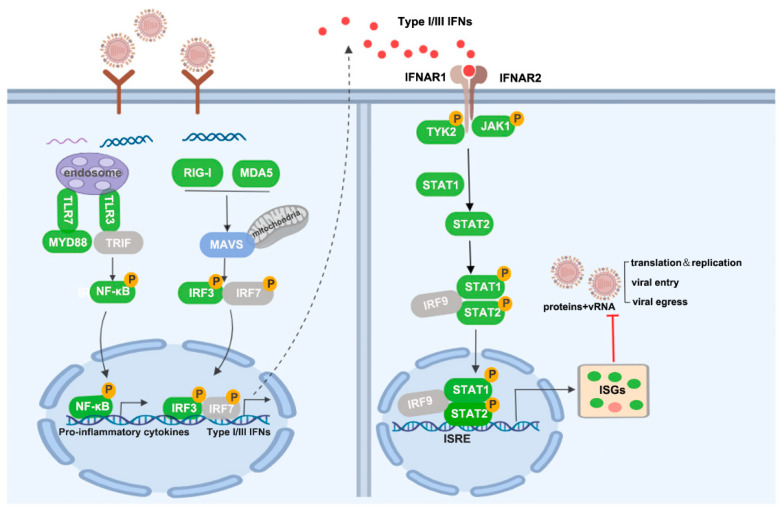
Schematic model of gene expression of sMDCK vs. aMDCK cells in the innate immune response against IAV infection. According to the transcriptome data, the green boxes represent downregulated genes, the red boxes represent upregulated genes, the blue boxes represent no significant change in gene expression, and the grey boxes represent not detected in gene expression (*p*-value < 0.05).

In summary, we analyzed the high-yield mechanism of sMDCK cells from the innate immune signaling pathway and found that downregulated ISGs promoted viral replication in sMDCK cells (Figure 9). Understanding the host innate immune responses to influenza infection in sMDCK cells will provide a scientific foundation for the genetic modification of MDCK cells and improve the production process of cell-based influenza vaccines.

## Figures and Tables

**Table 1:** Primers used for amplifying genes.

Primer Name	Sequence (5′ to 3′)
cRIG-I-F	ATGACGGCCGAGGAGCGG
cRIG-I-R	TCATTCGGGTATTTCTGGAGAAC
cIFIT3-F	ATGAGTGAGGTCAACAAGAATTC
cIFIT3-R	TCAGGTCTCAGGAGCTCTGAG
EGFP-F	ATGGTGAGCAAGGGCGAGGAG
EGFP-R	TTACTTGTACAGCTCGTCCATG

**Table 2:** Primers used for RT-qPCR.

Primer Name	Sequence (5′ to 3′)
cRIG-I mRNA-F	AGAGCAAGTTCAGTCAACTGTG
cRIG-I mRNA-R	ACTGAAGGTGGACATGGGTC
cSTAT1 mRNA-F	GGAACGTTCAGAGCACTGTG
cSTAT1-mRNA-R	ATGTTCTCGGTTCTGCAAGG
cIFIT3 mRNA-F	CTGGCCATAGCAATGTACTG
cIFIT3 mRNA-R	CTCTGTAATTTCAGGGCCAAG
cMAVS mRNA-F	TACCAGAGTCCCTGGAAGAG
cMAVS mRNA-R	AGTCAGAGGGTTGACAGCTG
cOAS1 mRNA-F	TCTTCAGGCAAAGGCACGAC
cOAS1 mRNA-R	ACTCTGGACCTCAAAGTACAC
cOAS3 mRNA-F	CCATCCCCGGTCTGAACTTTG
cOAS3 mRNA-R	CTGCCCCAGAGCGTCAAAG
cIFNB mRNA-F	GAGCAACGACTTGCTTCGATC
cIFNB mRNA-R	CTGGAACTGGCGTGATTTCTC
cISG15 mRNA-F	CATGGCTGGGAACCTGACTG
cISG15 mRNA-R	GAGATCCCATCCTGCAGCAC
cGAPDH mRNA-F	CCAAGAGGGTCATCATCTCTGC
cGAPDH mRNA-R	TGCCGAAGTGGTCATGGATG
PR8 NP-F	CTCTTGTTCGCACCGGAATG
PR8 NP-R	GTTCCGATCATTGATCCCACG
BVR-26 NP-F	CAGAGATAAAGAAGAGCGTCTAC
BVR-26 NP-R	TTCTTGTCATCAGTGGCAGC

## Data Availability

The data presented in this study are available on request from the corresponding author. The data are not publicly available due to security restrictions.

## Supplementary Materials

The following supporting information can be downloaded at: https://www.mdpi.com/article/10.3390/vaccines12030287/s1, Figure S1: Flowcytometric assay of α-2,3 and α-2,6 SA receptor in aMDCK and sMDCK cells.
