# Supplementary material for: Reduction in Interferon-Stimulated Genes Contributes to High-Yield Production of Influenza Virus in Suspension MDCK Cells
Source: Vaccines (Basel). 2024 Mar 9;12(3):287. doi: 10.3390/vaccines12030287 (PMC10974347; doi:10.3390/vaccines12030287)
Supplement: Supplementary file 1 [file vaccines-12-00287-s001.zip › vaccines-2886912-supplementary.pdf]

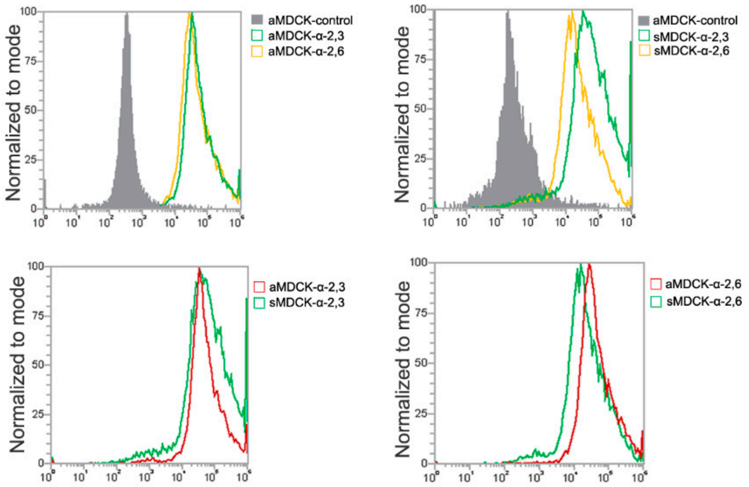

Supplementary Figure S1. Flowcytometric assay of α-2,3 and α-2,6 SA receptor in aMDCK and sMDCK cells.
